# Retroperitoneal Fibrosis: Still a Diagnostic Challenge

**DOI:** 10.7759/cureus.33998

**Published:** 2023-01-20

**Authors:** Sanchit Duhan, Bijeta Keisham, Kinza Bazigh, Chetna Duhan, Nasir Alhamdan

**Affiliations:** 1 Internal Medicine, Sinai Hospital of Baltimore, Baltimore, USA; 2 Radiodiagnosis, Smt Bhikhiben K Shah Medical Institute and Research Center, Sumandeep Vidyapeeth, Vadodara, IND

**Keywords:** rare autoimmune disease, igg 4 disease, alcoholic pancreatitis, rpf, : idiopathic retroperitoneal fibrosis

## Abstract

Retroperitoneal fibrosis (RPF) is a rare fibroinflammatory disorder usually involving the abdominal aorta and surrounding structures. It is divided into primary (idiopathic) and secondary RPF. Primary RPF can be immunoglobulin (Ig) G4-related disease or non-IgG4-related disease. Recently, there has been a rise in case reports regarding the topic, but awareness about the disease is still far from ideal. Hence, we present the case of a 49-year-old female who had repeated admissions for chronic abdominal pain attributed to chronic alcoholic pancreatitis. She had a medical history significant for psoriasis and surgical history significant for cholecystectomy. Her computed tomography (CT) scans on each admission for the last year showed some signs of RPF, but it was never considered the primary etiology of her chronic symptoms. We also obtained magnetic resonance imaging (MRI) which did not show any underlying malignancy but showed the progression of her RPF. She was started on a steroid regimen, which significantly improved her symptoms. She was diagnosed with idiopathic RPF due to unclear etiology, although her underlying risk factors, including psoriasis, past surgeries, and pancreatitis-associated inflammation, were considered predisposing factors. Idiopathic RPF accounts for more than two-thirds of total cases of RPF. Patients with autoimmune diseases can overlap with other autoimmune disorders. For non-malignant RPF, medical management with 1mg/kg/day steroids is deemed effective. Still, there is a lack of prospective trials and consensus for guidelines on treating RPF. The follow-up involves laboratory tests, including erythrocyte sedimentation rate, C-reactive protein, and CT or MRI in an outpatient setting to identify treatment response and relapse. There is a need for more streamlined guidelines to diagnose and manage this disease.

## Introduction

Retroperitoneal fibrosis (RPF) is an inflammatory disorder characterized by fibrosis around the abdominal aorta (usually infrarenal). It can extend to surrounding arteries and other structures, commonly ureters and inferior vena cava [1,2]. This can be idiopathic but also can be secondary to some medications (like bromocriptine, ergotamine, methyldopa, hydralazine, etc.), malignancies (like carcinoid, Hodgkin’s, sarcoma, carcinoma, etc.), infections (like tuberculosis, histoplasmosis, etc.), radiotherapy and post-surgery [3]. Idiopathic RPF has an incidence of 0.1-1.3 cases per 100,000 persons per year, with onset usually in age 50-60 years, more commonly in females [4].

The pathophysiology of the disease is still unclear. However, it has been postulated that idiopathic RPF might be a part of a systemic autoimmune disorder causing aortitis and subsequent fibro-inflammatory response around the aorta [5].

In a literature review of PubMed-indexed relevant articles, the etiologies associated with RPF included many diseases, including idiopathic disease, lymphomas, carcinomas, and drugs. Still, there has been a focus on Immunoglobulin (Ig) G4-related disease as likely underlying etiology in many cases. Given the rarity of the disease and unclear etiology in many cases, we present this case to help build more clinical data. 

## Case presentation

A 49-year-old female with psoriasis, chronic alcohol use disorder, cirrhosis, and chronic pancreatitis presented with abdominal pain, nausea, and multiple episodes of vomiting for five days. Her epigastric abdominal pain was sharp, continuous, progressive, radiating to the right flank, and 10/10 in intensity with no aggravating and relieving factors and no association with food intake. She had multiple episodes of non-bloody, non-bilious emesis containing food particles. Her last alcoholic drink was one day before admission.

A review of systems was significant for left-sided chest pain for several months, which was intermittent, sharp, 5-6/10 in intensity, episodes lasting for one minute before self-resolving, non-radiating, and associated with palpitations. She also endorsed significant unmeasured weight loss over the year.

Her medical history was significant for episodic worsening of chronic abdominal pain requiring repeated hospitalization almost every month for one and a half years. In prior admissions, this pain was postulated to be secondary to chronic alcoholic pancreatitis, and the patient was prescribed pancrelipase, but her pain persisted. Her surgical history was significant for tubal ligation and cholecystectomy. Her family history was important for colonic cancer in her mother and paternal grandmother.

On admission, her vital signs were stable and physical examination was remarkable for bilateral fine tremors in the upper extremities, abdominal rigidity, and mild abdominal distension with negative Murphy’s sign. The Clinical Institute Withdrawal Assessment score was 28. Laboratory workup showed high anion gap metabolic acidosis (anion gap: 18 mmol/L, beta-hydroxybutyrate: 1.94 mmol/L) with hypokalemic hypochloremia (potassium: 2.6 mmol/L, chloride: 93 mmol/L), likely secondary to diabetic ketoacidosis and emesis. Liver enzymes showed elevated alkaline phosphatase (399 U/L), aspartate aminotransferase (169 U/L), gamma glutamate aminotransferase (1327 U/L), total bilirubin (2.1 mg/dl), and direct bilirubin (1.1 mg/dl). Alanine aminotransferase (26 U/L) and lipase (19 U/L) were normal. Hepatitis was ruled out by a negative antigen-antibody panel (hepatitis B core IgM, hepatitis B surface antigen, hepatitis A IgM, and hepatitis C antibody). Erythrocyte sedimentation rate (ESR) was also elevated at 28 mm/hr, and C-reactive protein (CRP) was normal (Table 1).

**Table 1:** Assessment scale and laboratory values on admission.

Scales and Chemistries	Patient’s values	Normal reference range
Clinical Institute Withdrawal Assessment Scale	28	0
Anion Gap	18 mmol/L	8-16 mmol/L
Beta-hydroxy butyrate	1.94 mmol/L	0.02-0.27 mmol/L
Potassium	2.6 mmol/L	3.4-4.5 mmol/L
Chloride	93 mmol/L	98-107 mmol/L
Alkaline phosphatase	399 U/L	45-117 U/L
Aspartate aminotransferase	169 U/L	0-37 U/L
Alanine aminotransferase	26 U/L	12-78 U/L
Gamma-glutamyl transferase	1327 U/L	5-85 U/L
Lipase	19 U/L	13-60 U/L
Total bilirubin	2.1 mg/dL	0-1 mg/dl
Direct bilirubin	1.1 mg/dL	0.0-0.2 mg/dl
Erythrocyte sedimentation rate	28 mm/hr	0-24 mm/hr
C-reactive protein	0.6 mg/dL	0.3-1.0 mg/dL

**Figure 1:**
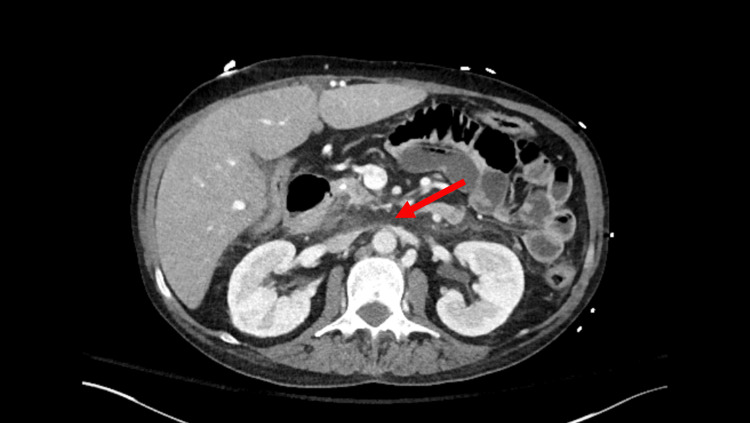
Computed tomography scan showing retroperitoneal fibrosis (red arrow).

Given her chest pain and emesis, a chest X-ray was obtained to look for any signs of mediastinitis, but it was unremarkable. A contrast-enhanced abdominal computed tomography (CT) was performed to look for acute pancreatitis, which showed stranding within the retroperitoneal fat extending throughout the abdomen and pelvis, including the perirectal and presacral regions (Figure 1). Also, multiple small retroperitoneal lymph nodes and larger nodes in gastrohepatic, para-aortic, and aortocaval spaces were seen.

**Figure 2:**
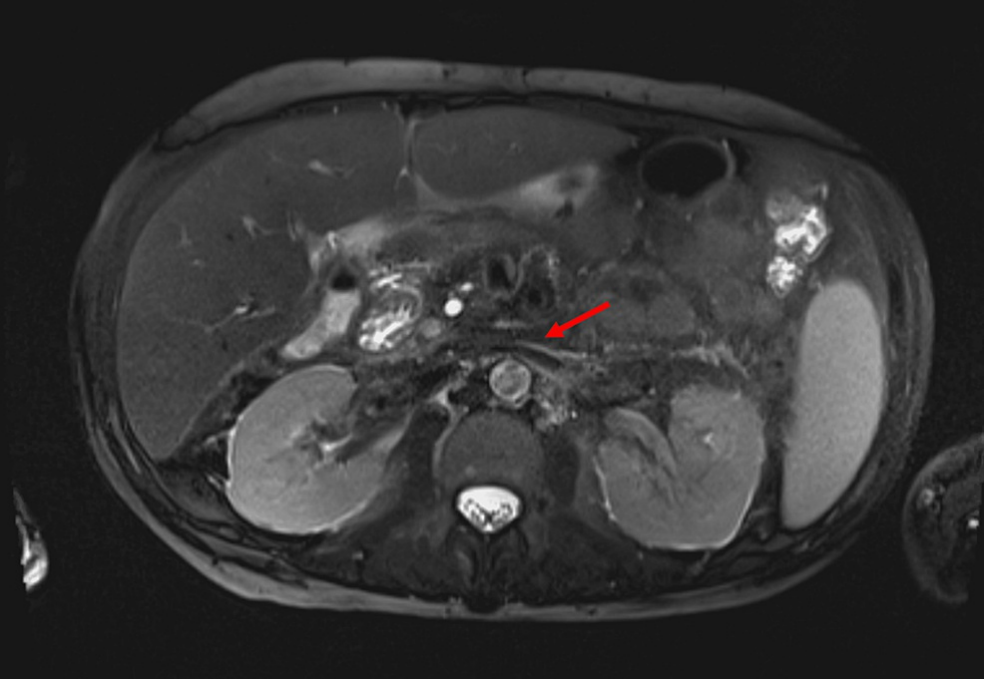
Magnetic resonance imaging showing retroperitoneal fibrosis (red arrow).

The patient’s abdominal pain was initially treated symptomatically with morphine. There was a suspicion of alcoholic chronic pancreatitis being the underlying etiology of abdominal pain associated with alcohol withdrawal. Gastroenterology was also consulted, and they recommended starting pancrelipase 24,000 units three times daily, pantoprazole, and thiamine supplementation. But due to the non-resolution of the patient’s pain and suspicion of underlying malignancy and biliary obstruction, a magnetic resonance imaging (MRI) accompanied by magnetic resonance cholangiopancreatography was performed which revealed retroperitoneal/periaortic fat stranding along the mesenteric vasculature with a noticeable gradual increase in multiple prior CTs in one year and multiple prominent cardio phrenic lymph nodes (Figure 2).

Given her repeated admissions for similar complaints, elevated ESR, and unclear etiology of RPF, a trial of steroids was deemed appropriate. Her symptoms improved significantly on steroid therapy, with a decline in ESR levels. She was diagnosed with idiopathic RPF and was started treatment on a long course of steroid therapy. She was discharged with education regarding the importance of outpatient follow-up to gauge treatment response and prevent relapse.

## Discussion

RPF can be idiopathic or secondary to other etiologies [3]. Idiopathic RPF accounts for more than two-thirds of total cases [6]. Idiopathic RPF can be IgG4-related or non-IgG4-related [7]. Twenty-five percent of patients with autoimmune disease can develop other autoimmune disorders [8]. Although extremely rare, cases of psoriasis associated with RPF have been seen in the past [9-11]. Unlike previous cases, our patient did not have an active flare-up of psoriasis, but an underlying autoimmune condition likely predisposed her to RPF. Idiopathic RPF has also been seen with autoimmune pancreatitis but not alcoholic pancreatitis [12]. Our patient lacked the imaging findings of autoimmune pancreatitis (sausage-shaped pancreas), and given the significant history of alcohol use; a biopsy was not warranted [13]. Whether alcoholic pancreatitis-associated inflammation played a role in RPF is uncertain. Our patient also had a history of abdominal surgeries, which might have contributed to the etiology [14].

The diagnosis of RPF remains a challenge, and like our patient, many others suffering from non-resolving abdominal pain with other abdominal comorbidities might be difficult to diagnose. RPF is commonly diagnosed with a CT or MRI. There has been discussion about the utility of 18F‑Fluorodeoxyglucose (18F‑FDG) positron emission tomography (PET) to identify extra-retroperitoneal lesions and associated underlying conditions. Still, given its low specificity, it does not provide a diagnostic role [4]. Idiopathic RPF is still considered a diagnosis of exclusion, and patients with RPF are recommended evaluation for other secondary etiologies before diagnosing them with idiopathic RPF. IgG levels are an essential indicator of IgG4-related RPF but are not enough for diagnosing IgG4-related disease and require a biopsy [15]. Other than IgG4-related RPF and underlying malignancy, the role of a biopsy in diagnosing RPF remains controversial and might be only beneficial if there is a lack of response to initial therapy or a lack of local experience with RPF [5]. 

Medical management with steroids and immunosuppressants is effective for non-malignant RPF [16]. Still, there is a lack of prospective trials and consensus for guidelines on treating RPF. Treatment response can be assessed with serial radiographic imaging in patients with non-IgG4 RPF [15]. The treatment requires a high dose of steroids, around 1mg/kg/day, for four to eight weeks [17]. A long course of steroids can be tapered once the remission is achieved. The follow-up involves laboratory tests, including ESR, CRP, and CT or MRI, at regular intervals in an outpatient setting to identify treatment response and relapse [17]. The effectiveness of steroid-sparing agents, including mycophenolate mofetil, methotrexate, cyclophosphamide, azathioprine, and tacrolimus, has been reported but not yet clinically proven. Rituximab, an anti-CD20 agent, has also been used to treat IgG4-related RPF [15].

Patients with ureteral obstruction often require surgical intervention along with steroid therapy. The initial approach includes symptomatic management with ureteral stent placement or percutaneous nephrostomy to relieve obstruction and preserve kidney function. But given the risks of infection and other complications associated with both procedures, they are removed as soon as patency is established on the pyelogram. Invasive surgical management with ureterolysis is required for patients who do not show improvement despite long-term steroid therapy. But given the rarity of the disease, expertise in the surgery is hard to achieve [15]. 

Even though RPF has been around for many decades, there is a need for more streamlined guidelines regarding diagnostic criteria, investigational approaches, and disease management.

## Conclusions

This case highlights the challenges faced in diagnosing RPF and emphasizes the role of clinical features and imaging in helping physicians achieve a diagnosis. It also hints towards the possibility of underdiagnosis of RPF in the current practice and the scope to increase awareness among physicians. As seen in our case, symptomatic patients can have severely reduced quality of life secondary to hindrances in their activities of daily living. There is a chance patients might remain undiagnosed despite multiple admissions, given the rarity of the disease, which can be detrimental to their physical and psychological health. Emphasis should be given to the likelihood of this diagnosis when a patient has underlying risk factors with unremitting abdominal pain. The underlying etiology should be investigated to search for any malignancy, and IgG4-related disease should be ruled out. Medical management involves a long course of steroid therapy. 
